# Expression of Bcl-2 and Bax in Mouse Renal Tubules during Kidney Development

**DOI:** 10.1371/journal.pone.0032771

**Published:** 2012-02-28

**Authors:** Xiao-Feng Song, Hao Ren, Arne Andreasen, Jesper Skovhus Thomsen, Xiao-Yue Zhai

**Affiliations:** 1 Department of Histology and Embryology, Institute of Pathology and Pathophysiology, China Medical University, Shen Yang, Liao Ning, China; 2 Department of Anatomy, Deaprtment of Biomedicine, Aarhus University, Aarhus, Denmark; 3 Department of Histology and Embryology, Liao Ning Medical College, Jin Zhou, Liao Ning, China; 4 Institute of Nephropathology, China Medical University, Shen Yang, Liao Ning, China; National Cancer Institute, United States of America

## Abstract

Bcl-2 and Bax play an important role in apoptosis regulation, as well as in cell adhesion and migration during kidney morphogenesis, which is structurally and functionally related to mitochondria. In order to elucidate the role of Bcl-2 and Bax during kidney development, it is essential to establish the exact location of their expression in the kidney. The present study localized their expression during kidney development. Kidneys from embryonic (E) 16-, 17-, 18-day-old mouse fetuses, and postnatal (P) 1-, 3-, 5-, 7-, 14-, 21-day-old pups were embedded in Epon. Semi-thin serial sections from two E17 kidneys underwent computer assisted 3D tubule tracing. The tracing was combined with a newly developed immunohistochemical technique, which enables immunohistochemistry on glutaraldehyde fixated plastic embedded sections. Thereby, the microstructure could be described in detail, and the immunochemistry can be performed using exactly the same sections. The study showed that Bcl-2 and Bax were strongly expressed in mature proximal convoluted tubules at all time points, less strongly expressed in proximal straight tubules, and only weakly in immature proximal tubules and distal tubules. No expression was detected in ureteric bud and other earlier developing structures, such as comma bodies, S shaped bodies, glomeruli, etc. Tubules expressing Bcl-2 only were occasionally observed. The present study showed that, during kidney development, Bcl-2 and Bax are expressed differently in the proximal and distal tubules, although these two tubule segments are almost equally equipped with mitochondria. The functional significance of the different expression of Bcl-2 and Bax in proximal and distal tubules is unknown. However, the findings of the present study suggest that the mitochondrial function differs between mature proximal tubules and in the rest of the tubules. The function of Bcl-2 and Bax during tubulogenesis still needs to be investigated.

## Introduction

The mechanisms involved in regulation of apoptosis have been studied extensively during the last decades. The caspase protein family has been reported to have a central role in apoptosis. The activation of the caspase proteins is regulated by a variety of factors including the B-cell lymphoma 2 (Bcl-2) protein family [1]. The anti-apoptosis protein Bcl-2 and the pro-apoptosis protein Bax are well known members of the Bcl-2 family, acting as opposed apoptosis regulators. Bcl-2 is associated with the outer mitochondrial membrane of viable cells and prevents Bax from perforating the outer mitochondrial membrane, causing cytochrome c to leak out [2]–[5]. When cytochrome c enters the cytoplasm, it binds to apoptotic protease-activating factor 1 (Apaf-1) leading to activation of caspase 9 and subsequently apoptosis [6], [7].

However, recently it has become evident that the function of the members of the Bcl-2 family is not limited to the regulation of apoptosis, but that they also play a role in the regulation of mitochondrial fusion and fission, and thereby in morphogenesis [8]–[10].

The kidney is a critical organ for filtering the plasma and is constitutively reabsorbing selected parts of the glomerular filtrate, which is an energy consuming process, located especially to the proximal and distal tubules. Therefore, the epithelial cells in these two tubule segments are rich in mitochondria. Kidney development is a complex process including basic morphogenesis of the collecting duct system and nephrons, involving interactions between the epithelium of ureteric buds and the metanephric mesenchyme [11], [12]. These processes are rigorously controlled in order to ensure that events happen at the right time and in the right sequence in order to build and maintain the various cell populations present in the different structures [13]–[16]. Thus, excessive or insufficient apoptosis during kidney development may cause anormogenesis [17], [18]. The expression of the Bcl-2 and caspase family members has been investigated *in vivo* and *in vitro* in order to explore their apoptotic and non-apoptotic function during kidney morphogenesis [19]–[20]. Furthermore, it has been suggested that members of the Bcl-2 and caspase families also are involved in cell adhesion, migration, differentiation, survival, and proliferation by interaction with other factors during kidney development [21]–[23]. However, the analysis of the apoptotic or non apoptotic roles of Bcl-2 and Bax during kidney development requires knowledge about the precise localization of their expression, which has not been available in previous studies in mice.

When inspecting the cells in e.g. a developing proximal tubule, the cells in one region may appear almost mature, arranged in one layer, and with a complete brush border, whereas the cells in the adjacent segment of the same tubule appears small and premature, and are arranged in cellular clusters. The cells in these clusters lack recognizable morphological features and their surface markers also make it difficult to classify them. This represents a gradient of maturation along the tubules in one direction, displaying different stages of cellular differentiation and development. Normally, such cell clusters can only be inspected out of their spatial morphological context, and their final destiny as e.g. proximal tubule cells will therefore be unknown. However, if the spatial morphological contex is known e.g. by tracing the tubules from a set of serial sections, we can connect and relate highly developed and less developed segments of the same tubule in space. Thereby, it is possible to bring the cell clusters into spatial context with the more developed part of the tubules.

Therefore, the aim of the present study was to perform computer assisted 3D tracing of the morphology of the developing kidney tubules combined with immunohistochemistry in order to localize the expression of Bcl-2 and Bax in developing kidneys in mice.

## Materials and Methods

### Animals

Kidneys from 9 prenatal and 18 postnatal Kunming mice were studied. The mice were maintained on standard specific-pathogen-free conditions. The day when the cervical mucus plug was observed was designated as embryonic day 0 (E0) and the day at birth was designated as postnatal day 0 (P0). Prenatal kidneys were obtained from E16, E17, and E18 fetuses, whereas postnatal kidneys were obtained from P1, P3, P5, P7, P14, and P21 pups. Three mice were obtained from separate litters for each time point. The animal experiments were performed in accordance with the code of Ethics of the World Medical Association (Declaration of Helsinki) and were approved by the Medical Ethics Committee of China Medical University.

### Antibodies

Rabbit polyclonal antibody against Bcl-2, mouse monoclonal antibody against Bax, FITC-labeled donkey antibody against rabbit, and TRITC-labeled donkey antibody against mouse were purchased from Abcam (Cambridge, CA).

### Preparation of renal tissues

The fetal kidneys were removed a few minutes after injection of pentobarbital sodium (50 mg/kg body weight) into the peritoneal cavity of the pregnant mice. The pup kidneys were preserved by perfusion fixation through the heart with a solution of 4% paraformaldehyde and 1% glutaraldehyde in 0.06 M sodium cacodylate buffer, pH 7.4. Subsequently, tissue blocks were cut perpendicular to the longitudinal axis of the kidneys and post-fixed for 1 h in 1% OsO_4_ in 0.1 M sodium cacodylate buffer. The tissue blocks were then dehydrated in ethanol and acetone and embedded in Epon 812 (TAAB, Aldermaston, Berks, UK). Then, approximately 700 2.5-µm-thick serial sections were obtained parallel to the axis through cortex to papilla from two of the three E17 mice and stained with toluidine blue. The E17 kidneys were selected for serial sectioning and subsequent computer assisted renal tubule tracing as the nephrons from the E17 kidneys represent all developing stages. Thus, it is possible to avoid the very time consuming and laborious image handling and 3D tracing of kidneys to all time points.

### Image recording and aligning for 3D tubule tracing

All toluidine blue stained sections from the two serial sectioned E17 kidneys were digitized using a microscope (Olympus AX70, Tokyo, Japan) equipped with a motorized stage and a digital camera (Olympus DP50, Tokyo, Japan) attached to a standard PC. For each section four partly overlapping digital images were recorded and combined into one 24-bit color image using analySIS (version 3.2, Soft Imaging System, Münster, Germany). The final image size was 2700×2000 pixels resulting in an isotropic pixel size of 0.93 µm. The digitized images from the two E17 mice were aligned into two image stacks using a custom-made computer program running under Linux (openSUSE 11.2, www.opensuse.org). The image aligning program automatically rotates and translates two consecutive images in order to minimize the difference between them. The function that seeks to minimize the difference between the two images uses a gradient-based method. However, there is a chance for this method to be trapped in a local minimum as the tubular structure of the kidney exhibit a large degree of translation symmetry. In order to avoid this, 30 different sets of initial conditions were selected using a Monte Carlo method for each image pair and the resulting image transformation values providing the best fit was chosen for the aligning. Subsequently, the transformation values were high pass filtered in order to avoid slow drift of the image stack before being used for the final image transformations [24], [25].

### Digital tracing of the renal tubules

**Figure 1:**
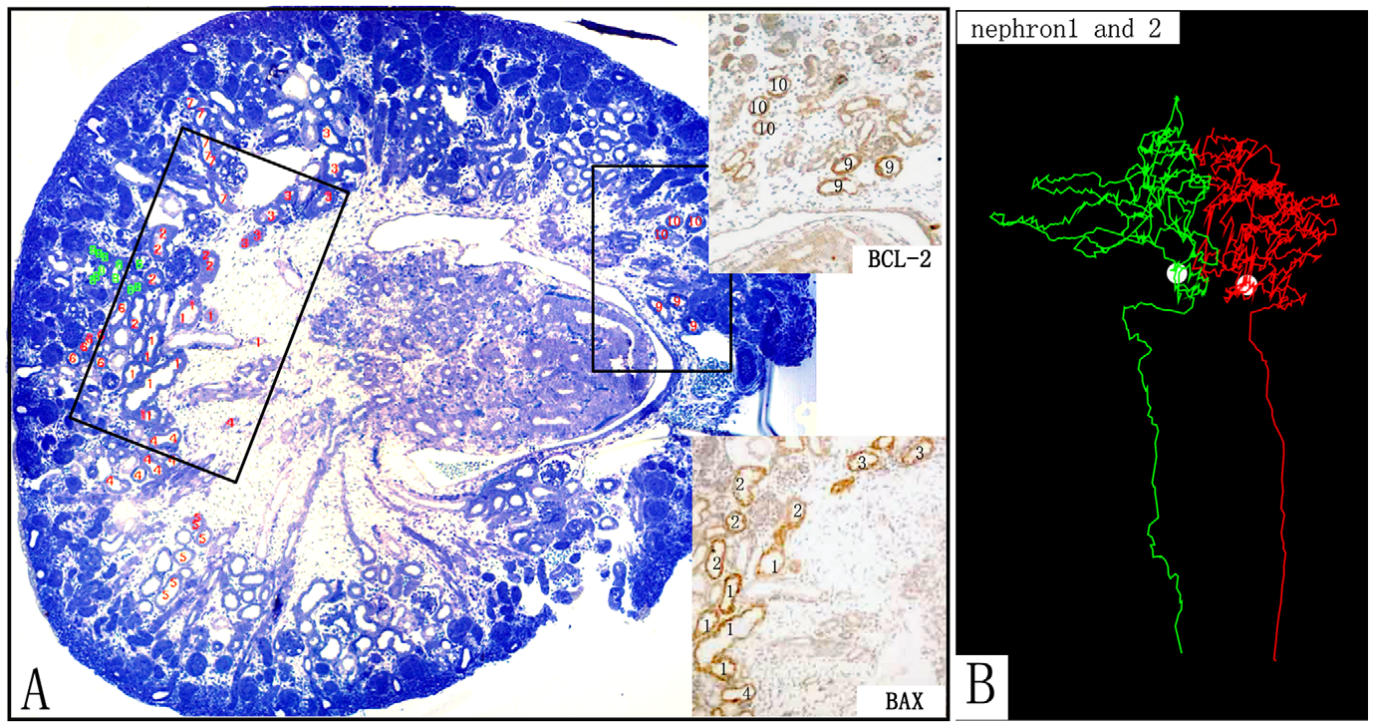
Histological image and 3D reconstructions of one of the E17 kidneys. A) Image of one of the serial sections from one of the E17 kidneys. The red and green figures represent the tubules, which have been traced and are presently being traced, respectively. The expression of Bcl-2 (upper) and Bax (lower) derived from the same section is localized to proximal tubules as determined by the tracings. B) Two 3D reconstructions of proximal convoluted tubules and descending limbs. The two nephrons are numbered 1 and 2 in A.

The spatial course of the renal tubules was traced with a custom-made computer program running on the Linux-based PC as previously described in detail [25]. In brief: The aligned image stack was interactively viewed on a computer screen and the *x*-, *y*-, and *z*-coordinates along the courses of the renal tubules were demarcated with a computer mouse and recorded in a data file (Figure 1A). Subsequently, this data was analyzed and visualized in order to investigate the spatial relationship between the traced tubules.

### Immunohistochemistry

Selected sections from the two E17 kidneys used for 3D tracing were re-embedded and two 1-µm-thick consecutive sections were obtained from each of the 2.5-µm-thick sections. One-µm-thick semi-thin sections were obtained from all the other kidneys at all time points. The sections were pretreated with Maxwell solution (20 pellets of KOH in 10 ml absolute methanol and 5 ml propylene oxides) in order to remove the epoxy material and were then heated in a microwave oven in order to retrieve the antigen as previously described in detail [26].

For localization of Bcl-2 and Bax with light microscopy the sections were incubated with either rabbit anti-Bcl-2 IgG (1∶200) or mouse anti-Bax IgG (5 µg/ml), followed by incubation with peroxidase-conjugated secondary anti-rabbit and anti-mouse antibodies respectively (1∶200). The peroxidase was visualized with diaminobenzidine (DAB). Thereafter, the sections were counterstained with haematoxylin for less than 2 minutes, washed in running water, dehydrated in graded ethanol, soaked in xylene, and mounted with Eukitt. The sections were examined with a light microscope (Nikon 80iBF-F-P, Tokyo, Japan) equipped with a Nikon DS digital camera.

For co-localization of Bcl-2 and Bax with confocal microscopy the sections were incubated with a mixture of rabbit anti-Bcl-2 (1∶200) and anti-Bax (5 µg/ml), followed by incubation with secondary anti-rabbit-FITC IgG (1∶600) and anti-mouse-TRITC IgG (1∶300). After the sections were washed in PBS, they were mounted with glycerol. The sections were examined with a confocal microscope (Nikon CI plus, Tokyo, Japan).

For all immunohistochemical studies, control sections were incubated in PBS buffer instead of the primary antibodies.

## Results

### 3D morphology of the developing kidney

The computer assisted 3D nephron tracing made it possible to define and localize the various structures of the E17 kidneys. In the E17 kidneys, the renal structures at all developing stages were observed. In all, 46 tubules were traced in the two E17 kidneys, including 19 ureteric buds and 25 mature (juxtamedullary nephrons) and immature nephrons.

Various sections of the terminal ureteric buds were observed in the superficial cortex. Condensed stroma cells, renal vesicles, and comma-shaped bodies were observed around the tip of the ureteric buds. S-shaped bodies as well as developing corpuscles and developing tubules were located in the mid-cortex to the juxta-medullary cortex. The proximal straight tubules and their corresponding distal straight tubules from the maturing corpuscles were found to extend into or from the middle area (original medulla) of the kidney in bundles surrounded by a few developing vessels and collecting ducts. The spatial course of the proximal tubules in the juxta-medullary cortex resembled that of the adult mature proximal tubules (Figure 1).

The renal structures at other developing time points were identified by comparing them with the traced tubules from the E17 kidneys and from adult kidneys [25].

### Localization of Bcl-2 and Bax

**Figure 2:**
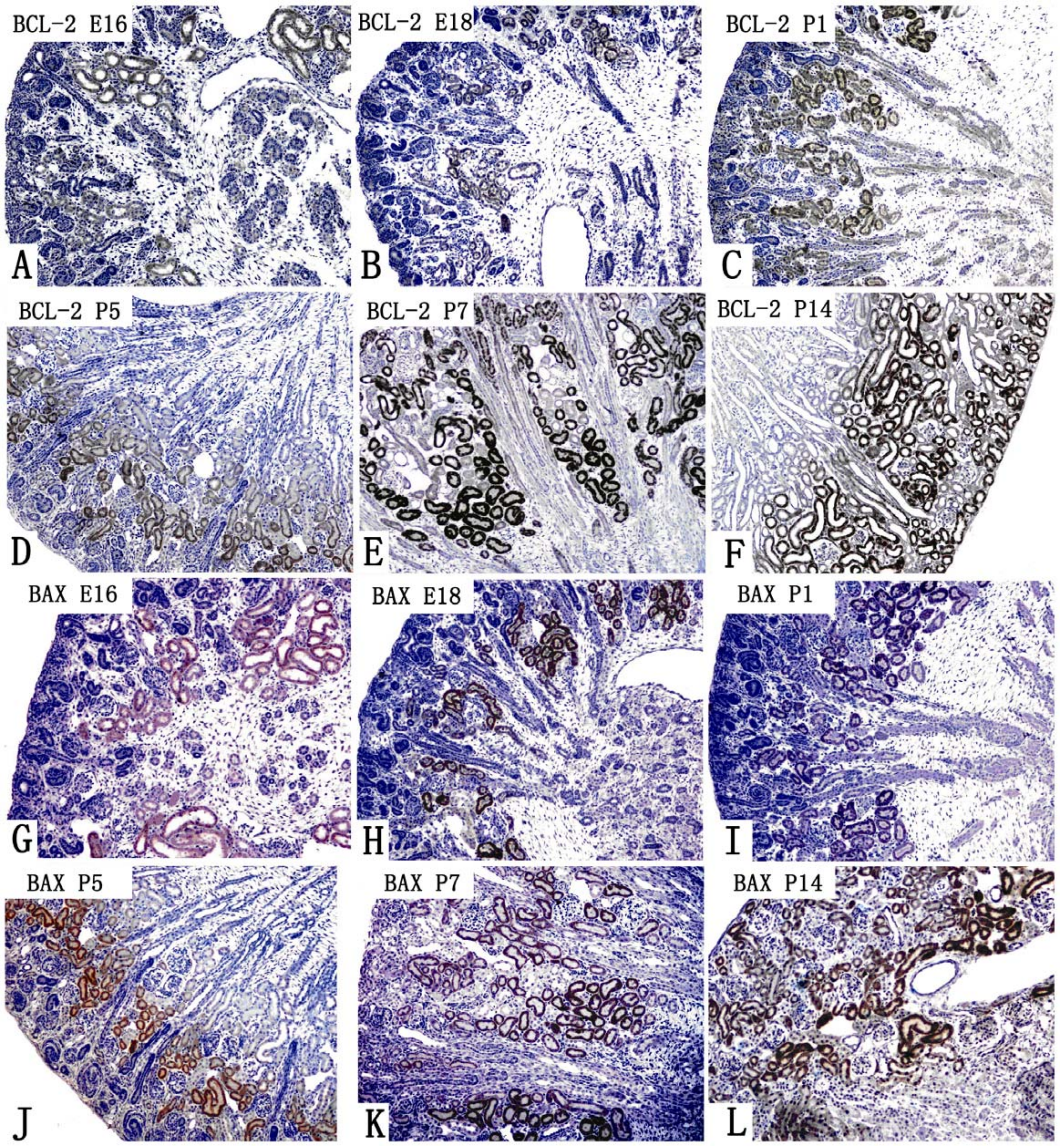
Expression of Bcl-2 (A–F) and Bax (G–L) in developing mouse kidneys. Strong expression of Bcl-2 and Bax are localized to mature proximal tubule cells at all time points, and the area of the expression was mainly in the cortical labyrinth, gradually expanding from the juxta-medullary cortex to the middle and superficial cortex, corresponding to the maturation of the proximal tubules in the cortical labyrinth. The proximal tubules in the medullary rays and medulla were weakly expressing Bcl-2 and Bax at the later stages of kidney development.

The mature proximal tubule cells showed a strong positive reaction for both Bcl-2 (Figure 2A–F) and Bax (Figure 2G–L) at all time points. The strongest expression was found in the mature proximal convoluted tubules in the juxta-medullary cortex (Figure 1). Less strong expression was found in the proximal straight tubules in both the medullary rays (at P7) and the medulla. Only weak expression was found in immature proximal tubules and in distal tubules. In contrast, no expression of Bcl-2 and Bax was detected in ureteric buds and other early developing structures, such as comma bodies, S shaped bodies, glomeruli, etc. at any time point. Thus, with development of the kidney, the area with strong expression of both Bcl-2 and Bax gradually expand from the juxta-medullary cortex to the mid-cortex, and eventually to the entire cortex, corresponding to the area of maturation of the proximal tubules in the cortex.

### Collocalization of Bcl-2 and Bax

**Figure 3:**
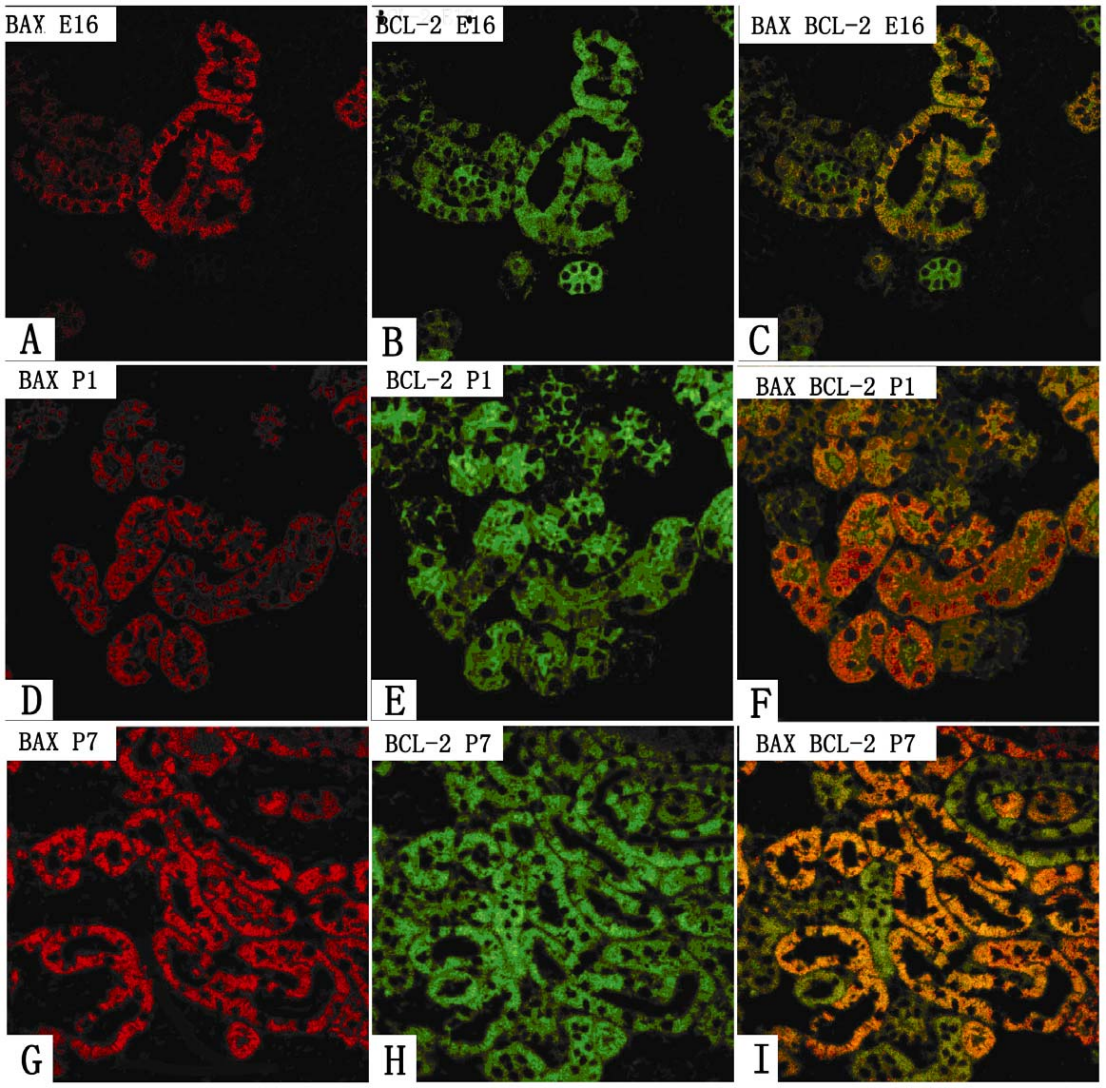
Co-localization of Bcl-2 and Bax using double labeled immunofluorescence in the developing kidneys. Green stained tubules represent Bcl-2, red stained tubules represent Bax, while yellow stained tubules represent co-expression of Bcl-2 and Bax. Tubules that are expressing Bcl-2 only are occasionally seen.

The investigation of the sections double labeled for both Bcl-2 and Bax showed that most of the tubules that expressed Bax also expressed Bcl-2 except for some tubules that expressed Bcl-2 only (Figure 3).

## Discussion

It has recently been established that Bcl-2 and Bax not only play a role in apoptosis, but are also involved in kidney morphogenesis and mitochondrial morphogenesis. In order to elucidate which role Bcl-2 and Bax play during kidney development, it is essential to establish the exact location of their expression in the kidney. However, it is difficult to identify the different tubule segments at the different stages in the developing kidney from antigens expressed by the epithelial cells without the morphological context. Therefore, we employed computer assisted tubular tracing so that the renal morphology could be combined with immunohistochemical identification of Bcl-2 and Bax. For tubule tracing, the kidneys were fixed with glutaraldehyde and osmium tetroxide, and embedded in plastic in order to preserve the kidney morphology. However, it is well known that such tissue preparation procedures weaken the tissue antigenicity, as aldehyde fixatives cross-link with the tissue antigens and as the epoxy resin mask the antigens [27]. Therefore, we applied a newly developed technique, which enables immunohistochemistry on glutaraldehyde fixated plastic embedded sections by etching the epoxy material and retrieving the antigens [26]. In this way we could perform immunohistochemistry using exactly the same sections as used for the establishing the morphology through the tubule tracing. Thus, this technique allowed us an unprecedented ability to pinpoint the spatial location of the expression of Bcl-2 and Bax in developing kidneys.

By selecting the E17 kidneys for the computer assisted 3D tracings we ensured that all of the different stages of the developing nephrons could be observed. The 3D reconstructions of the E17 kidneys could then be used as a guide for identifying all the different tubules in the developing kidneys at the other time points.

The results showed that the expression of Bcl-2 and Bax was most pronounced in the convoluted part of the mature proximal tubules, whereas the expression of Bcl-2 and Bax was less pronounced in the straight tubules running in medullary rays and in the medulla. Only weak expression was found in immature proximal tubules and in distal tubules. No expression was detected in ureteric buds and other early developing structures, such as comma bodies, S shaped bodies, glomeruli, etc.

It has previously been established that Bcl-2 and Bax are involved in the regulation of apoptosis [2]–[5], as well as in mitochondrial fusion and fission [8]–[10]. Since the amount of mitochondria is similar in proximal and distal tubules, the difference in expression of Bcl-2 and Bax might be attributable to functional differences between proximal and distal tubule mitochondria. This view is supported by a recent study, which revealed functional differences between mitochondria from proximal and distal tubule cells (thick ascending limbs) [28]. These differences were mainly observed in mitochondrial membrane potential, the expression ratio of ATPase and its inhibitor, the product of reactive oxygen species, and glutathione levels. Furthermore, mitochondrial glycolysis differs between proximal and distal tubule cells, and between proximal convoluted and straight tubule cells. This is supported by the findings of a series of *in vitro* studies, which show that the glycolysis in the S3 segment of the proximal tubule is less pronounced than in the distal tubules [29], [30]. All these functional differences between proximal and distal tubule mitochondria may explain why the proximal tubule is more vulnerable to ischemic, hypoxic, and toxic injury in adulthood than the distal tubule [13], [31]–[34]. Thus, damage to the distal tubule is only observed after severe renal ischemia reperfusion injury [35]. It has previously been suggested that Bcl-2 and Bax plays a pivotal role in inducing mitochondria-mediated apoptosis in glomerular diseases and ischemic and toxic kidney injury [36]. Therefore, it is interesting that we found that Bcl-2 and Bax were mainly co-expressed in mature proximal convoluted tubules. If such an ischemia initiates an apoptotic response and if the tissue damage continues after the circulation has been reestablished, then it might be possible to reduce tissue damage by interacting with the Bcl-2 and Bax apoptosis regulating activity. However, at present it is too early to suggest the existence of such a treatment of acute renal failure. Thus, our findings warrant further investigation of in vitro and in vivo models to investigate such potential treatments.

Furthermore, our findings agree with an observed enhanced expression of Bcl-2 and Bax in regenerating proximal tubule cells [37].

Apoptosis is believed to depend on a relatively high level of Bax compared to Bcl-2. Consequently, co-expression of Bcl-2 and Bax is not necessarily a marker for apoptosis itself since Bcl-2 and Bax may counteract each other.

Previous studies has shown that Bcl-2 deficiency results in kidney maldevelopment [38], [39], which illustrates that the Bcl-2 family members are important for the morphological development of the kidney. The present study has for the first time mapped the spatial location of Bcl-2 and Bax expression during kidney development, which is the first step in understanding the role of these proteins during kidney development.

In conclusion: The tracing of tubules using serial sections combined with immunohistochemistry of the same sections has enabled us – for the first time – to localize the expression of Bcl-2 and Bax in the different renal structures of the developing kidney. We found a strong co-expression of Bcl-2 and Bax in mature proximal convoluted tubules, which is the tubule segment that is the most vulnerable to ischemia, whereas the expression of Bcl-2 and Bax in straight tubules was less pronounced. Only weak expression Bcl-2 and Bax was found in immature proximal and distal tubules. No expression of Bcl-2 and Bax was detected in ureteric buds and other earlier developing structures, such as comma bodies, S shaped bodies, glomeruli, etc. The functional significance of the different expression of Bcl-2 and Bax in proximal and distal tubules is unknown. However, the findings of the present study suggest that the mitochondrial function differs between mature proximal tubules and in the rest of the tubules. The function of Bcl-2 and Bax during tubulogenesis still needs to be investigated.

## References

[1] Wyllie AH (2010). “Where, O Death, Is Thy Sting?” A Brief Review of Apoptosis Biology.. Mol Neurobiol.

[2] Kuwana T, Mackey MR, Perkins G, Ellisman MH, Latterich M (2002). Bid, Bax, and lipids cooperate to form supramolecular openings in the outer mitochondrial membrane.. Cell.

[3] Mikhailov V, Mikhailova M, Pulkrabek DJ, Dong Z, Venkatachalam MA (2001). Bcl-2 prevent Bax oligomerization in the mitochondrial outer membrane.. J Biol Chem.

[4] Yang J, Liu X, Bhalla K, Kim CN, Ibrado AM (1997). Prevention of apoptosis by Bcl-2: release of cytochrome c from mitochondria blocked.. Science.

[5] Marchetti P, Castedo M, Susin SA, Zamzami N, Hirsch T (1996). Mitochondrial permeability transition is a central coordinating event of apoptosis.. J Exp Med.

[6] Li P, Nijhawan D, Budihardjo I, Srinivasula SM, Ahmad M (1997). Cytochrome c and dATP-Dependent Formation of Apaf-1/Caspase-9 Complex Initiates an Apoptotic Protease Cascade.. Cell.

[7] Riedl SJ, Li W, Chao Y, Schwarzenbacher R, Shi Y (2005). Structure of the apoptotic protease-activating factor 1 bound to ADP.. Nature.

[8] Rolland SG, Conradt B (2010). New role of the BCL-2 family of proteins in the regulation of mitochondrial dynamics.. Curr Opin Cell Biol.

[9] Autret A, Martin SJ (2009). Emerging role for members of the Bcl-2 family in mitochondrial morphogenesis.. Mol Cell.

[10] Westermann B (2010). Mitochondrial fusion and fission in cell life and death.. Nat Rev Mol Cell Biol.

[11] Costantini F (2006). Renal branching morphogenesis:concepts, questions, and recent advances.. Differentiation.

[12] Walker KA, Sims-Lucas S, Caruana G, Cullen-McEwen L, Li J (2011). Betaglycan is required for the establishment of nephron endowment in the mouse.. PloS One.

[13] Hammerman MR (1998). Growth factors and apoptosis in acute renal injury.. Curr Opin Nephrol Hypertens.

[14] Clarke PG (1990). Developmental cell death: morphological diversity and multiple mechanisms.. Anat Embryol.

[15] Carev D, Krnić D, Saraga M, Sapunar D, Saraqa-Babic M (2006). Role of mitotic, pro-apoptotic and anti- apoptotic factors in human kidney development.. Pediatr Nephrol.

[16] Foley JG, Bard JB (2002). Apoptosis in the cortex of the developing mouse kidney.. J Anat.

[17] Torchinsky A, Savion S, Gorivodsky M, Shepshelovich J, Zaslavsky Z (1995). Cyclophosphamide-induced teratogenesis in ICR mice the role of apoptosis.. Teratog Carcinog Mutagen.

[18] Chen B, Cyr DG, Hales BF (1994). Role of apoptosis in mediating phosphamide mustard-induced rat embryo malformation in vitro.. Teratolgy.

[19] Hayashi M, Araki T (2002). Caspase in renal development.. Nephrol Dial Transplant.

[20] Novack DV, Korsmeyer SJ (1994). Bcl-2 protein expression during murine development.. Am J Pathol.

[21] Sheibani N, Scheef EA, Dimaio TA, Wang Y, Kondo S (2007). Bcl-2 expression modulates cell adhesion and migration promoting branching of ureteric bud cells.. J Cell Physio.

[22] Ziehr J, Sheibani N, Sorenson CM (2004). Alterations in cell-adhesive and migratory properties of proximal tubule and collecting duct cells from bcl-2−/−mice.. Am J Physiol Renal Physiol.

[23] Lamkanfi M, Festjens N, Declercq W, Vanden Berqhe T, Vandenabeele P (2007). Caspases in cell survival, proliferation and differentiation.. Cell Death Differ.

[24] Andreasen A, Ren H (2003). Extending the resolution of light microscopy digitized images with reference to cellular changes after in vivo low oxygen exposure.. J Neurosci Methods.

[25] Zhai XY, Thomsen JS, Birn H, Kristoffersen IB, Andreasen A (2006). Three-dimensional reconstruction of the mouse nephron.. J Am Soc Nephrol.

[26] Zhai XY, Kristoffersen IB, Christensen EI (2007). Immunocytochemistry of renal membrane proteins on epoxy sections.. Kidney Int.

[27] Kellenberger E, Durrenberger M, Villiger W, Carlemalm E, Wurtz M (1987). The efficiency of immunolabel on lowicryl sections compared to theoretical predictions.. J Histochem Cytochem.

[28] Hall AM, Unwin RJ, Parker N, Duchen MR (2009). Multiphoton imaging reveals differences in mitochondrial function between nephron segments.. J Am Soc Nephrol.

[29] Ruegg CE, Mandel LJ (1990). Bulk isolation of renal PCT and PST.II. Differential responses to anoxia or hypoxia.. Am J Physiol.

[30] Uchida S, Endou H (1988). Substrate specificity to maintain cellular ATP along the mouse nephron.. Am J Physiol.

[31] Kim YJ, Kim YA, Yokozawa T (2009). Protection against oxidative stress, inflammation, and apoptosis of high-glucose-exposed proximal tubular epithelial cells by astaxanthin.. J Agric Food Chem.

[32] Messaris E, Memos N, Chatzigianni E, Kataki A, Nikolopulou M (2008). Apoptotic death of renal tubular cells in experimental sepsis.. Surg Infect.

[33] Patschan D, Michurina T, Shi HK, Dolff S, Brodsky SV (2007). Normal distribution and medullary-to -cortical shift of Nestun-expressing cells in acute renal ischemia.. Kidney Int.

[34] Saikumar P, Dong Z, Patel Y, Hall K, Hopfer U (1998). Role of hypoxia- induced Bax translocation and cytochrome c release in reoxygenation injury.. Oncogene.

[35] Arany I (2008). When less is more: apoptosis during acute kidney injury.. Kidney Int.

[36] Erkan E, Devarajan P, Schwartz GJ (2007). Mitochondria are the major targets in albumin-induced apoptosis in proximal tubule cells.. J Am Soc Nephrol.

[37] Basile DP, Liapis H, Hammerman MR (1997). Expression of bcl-2 and bax in regenerating rat renal tubules following ischemic injury.. Am J Physiol.

[38] Sorenson CM (2004). Bcl-2 family members and disease.. Biochim Biophys Acta.

[39] Fedorov LM, Schmittwolf C, Amann K, Thomas W-H, Müller AM (2006). Renal failure causes early death of bcl-2 deficient mice.. Mech Ageing Dev.

